# Chiral Nematic Cellulose Nanocrystal Films Cooperated with Amino Acids for Tunable Optical Properties

**DOI:** 10.3390/polym13244389

**Published:** 2021-12-15

**Authors:** Xiao Xiao, Jie Chen, Zhe Ling, Jiaqi Guo, Jianbin Huang, Jianfeng Ma, Zhi Jin

**Affiliations:** 1 Key Lab of Bamboo and Rattan Science & Technology, International Center for Bamboo and Rattan, Beijing 100102, China; xiao_xiao@pku.edu.cn (X.X.); majf@icbr.ac.cn (J.M.); 2 Beijing National Laboratory for Molecular Sciences (BNLMS), State Key Laboratory for Structural Chemistry of Unstable and Stable Species, College of Chemistry and Molecular Engineering, Peking University, Beijing 100871, China; jbhuang@pku.edu.cn; 3 Jiangsu Co-Innovation Center of Efficient Processing and Utilization of Forest Resources, College of Chemical Engineering, Nanjing Forestry University, Nanjing 210037, China; chenjielalala@njfu.edu.cn (J.C.); jiaqi.guo@njfu.edu.cn (J.G.); 4 Research Institute of Wood Industry, Chinese Academy of Forestry, Beijing 100091, China

**Keywords:** cellulose nanocrystal films, chiral nematic structure, amino acids, nanocomposites, optical properties

## Abstract

The exploration of functional materials relies greatly on the understanding of material structures and nanotechnologies. In the present work, chiral nematic cellulose nanocrystal (CNC) films were prepared by incorporation with four types of amino acids (AAs, glycine, histidine, phenylalanine, and serine) via evaporation-induced self-assembly. The films present ideal iridescence and birefringence that can be tuned by the amount of AAs added. The intercalation of AAs enlarged the pitch values, contributing to the red-shift trend of the reflective wavelength. Among the AAs, serine presented the most compatible intercalation into cellulose crystals. Interestingly, histidine and phenylalanine composite films showed high shielding capabilities of UV light in diverse wavelength regions, exhibiting multi-optical functions. The sustainable preparation of chiral nematic CNC films may provide new strategies for materials production from biocompatible lignocellulose.

## 1. Introduction

Cellulose is one of the most abundant organic materials in the biosphere. With a linear polysaccharide linked by β-1,4-glycosidic bonds of D-glucose, cellulose presents extremely high potential in bioconversion and the preparation of functional polymer materials [1,2,3]. Cellulose nanocrystals (CNCs) are existing domains of cellulose polymers and can be separated via hydrolysis or enzymatic hydrolysis from the distinctive two-phase structure containing ordered crystalline and amorphous parts [4,5,6]. Rod-shaped CNCs exhibit ideal biocompatibility, sustainability, high strength and modulus, controllable structure, and feasible surface modification [7,8,9]. More interestingly, CNC suspensions can self-assemble into chiral nematic phases at critical colloidal concentration [10]. Meanwhile, the left-handed nematic phase can be further used for solid film preparation. The CNC films prepared by evaporation-induced self-assembly (EISA) reflect left-handed circularly polarized (LCP) light due to their left-handed nanostructure [11]. Visibly, the films present birefringence and iridescence, and it can be tuned by a photonic band-gap that is also called the pitch [12]. As green and advanced multi-functional nanomaterials, CNC-based iridescent films have attracted great attention among researchers [13].

Based on the above properties, chiral nematic CNC films have shown great potential in many fields such as sensors, encryptors, light shutters, and templates [14]. Large-scale preparation of chiral nematic CNC films has also been reported recently [15]. During the exploration of CNC film applications, some shortcomings are revealed. For example, due to the high rigidity of CNCs, the film may exhibit brittleness and limited toughness. The problem can be solved by incorporating other composites as fillers, such as glycerol [16], PEG [17], PVA [18], and even some amorphous polysaccharides [10]. Via a series of modifications, CNC-based biocomposites possess a good balance of controlled color appearances in a wide spectral range and improved mechanical performance as compared to traditional CNC-based synthetic polymer composites. More simply, researchers have reported a combination of CNCs and glucose for facile film preparation. As a result, close binding with glucose induces the rearrangement of a CNC chain and strengthens the repulsive interaction, thus increasing the helical pitch of the chiral nematic structure and changing the macroscopic color of the films [19]. Together with the improvement of mechanical strength, the films demonstrate a reversible structural color change between blue and red at a relative humidity (RH) between 50% and 98% [20].

More recently, with increasing attention on biomimetic and biocompatible materials, CNC films incorporated with the main component of living bodies, proteins or amino acids (AA), have received great interest [21]. CNC/chitin/silk fibroin composite films displayed obvious iridescent color, which can shift from blue to yellow-red corresponding to the higher concentrations [22]. The mechanical properties and thermal stabilities were also improved simultaneously. With the combination of D- or L-histidine, packing and crystallization of AA were revealed on the CNCs due to the surface charge. However, the different conformation of D-histidine shows heavy disturbance during the self-assembly of the films [23]. Moreover, CNCs have been infiltrated at various loadings with silk proteins and bovine serum albumin to fabricate composite films. A considerably higher toughness was observed for chiral nematic CNC films infiltrated with the denatured silk fibroin, compared to the folded one [24].

The research on CNCs–protein and CNCs–AA has shown some interesting trends, such as a tunable iridescent phenomenon, mechanical properties, and thermal stabilities. However, there are still some remaining problems, including the rules of the polarized chirality, comparisons between different AA compositions, as well as their contributions to the promoted applicable functions of the films. In this study, four types of AA (glycine, histidine, phenylalanine, and serine) were individually incorporated with CNCs at various ratios to prepare composite films via EISA. A series of optical, microstructural, and chemical properties were characterized. Meanwhile, other functions besides the iridescence were also explored in order to search for broader fields for the utilization of sustainable CNC composite films.

## 2. Materials and Methods

### 2.1. Materials

Softwood pulp was used to prepare CNC suspensions by sulfuric acid hydrolysis. Approximately 2 g of cellulose were added to 100 mL of 55 wt.% sulfuric acid at 60 °C for 1.5 h with mechanical stirring (400 rpm). The dispersion was diluted four-fold in water, followed by rinsing with three repeated centrifuge cycles. Afterwards, samples were dialyzed against deionized water for several days, until the pH reached ∼6 to disperse the cellulose [25]. Amino acids (AA) including histidine (His), phenylalanine (Phe), glycine (Gly), and serine (Ser) were purchased from Macklin Chemical Reagent (Shanghai, China).

### 2.2. Modification and Preparation of CNCs Film

The AAs were individually dissolved in deionized water to prepare the solution with a solids content of 1%. To make the hybrid CNCs–AA solution, CNCs dispersion (0.98%) and AA solution (1%) were mixed. The solids ratios of CNCs and AA were, respectively, 100:0, 95:5, 90:10, 85:15, 80:20, and 70:30. Then, the mixture solutions were magnetically stirred at room temperature for 10 h. After that, these suspension series were individually cast in a 60 mm-diameter dish (5 mL) and two 30 mm-diameter dishes (2.5 mL) to form films. The sample of 100:0 was referred to as pure CNC, and for the other CNC ratios, from high to low, the samples were separately denoted as G1–G5 (glycine), H1–H5 (histidine), P1–P5 (phenylalanine), and S1–S5 (serine).

### 2.3. Characterizations

All the samples’ transmittances were tested using a UV–Vis spectrometer (UV-1800, Shimadzu, Kyoto, Japan). The incorporated CNCs–AA suspensions were observed using a JSM-1400 transmission electron microscope (JEOL, Tokyo, Japan). Carl Zeiss (Axio Observer A1) inverted microscope equipped with crossed polarizers was used for polarized optical microscopy (POM, Zeiss, Oberkochen, Germany) analysis. Scanning electron microscopy (SEM) was performed using JSM-7600F (JEOL, Tokyo, Japan) at an accelerating voltage of 3 kV. The samples were coated with a thin layer of gold before characterization. For chemical characterizations, the films were analyzed using a Nicolet 6700 infrared spectrophotometer (IR, Thermo Fisher Scientific, Cleveland, OH, USA) equipped with an ATR accessory (ATR-IR). The crystal structure of the films was tested using a Rigaku Ultima IV X-ray diffractometer (Rigaku, Tokyo, Japan) with Cu Kα-radiation (λ = 0.15419 nm).

## 3. Results and Discussion

### 3.1. EISA of the Composite Films

**Figure 1:**
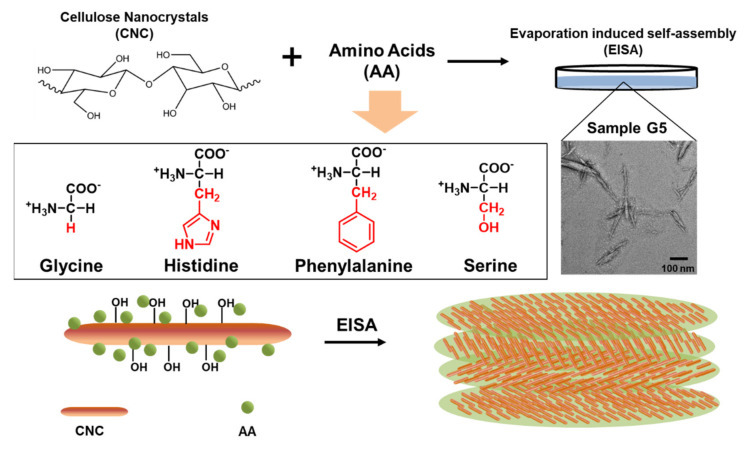
The scheme of the CNCs–AA composite films preparation: the molecular structures of four types of AAs; TEM image of sample G5 suspensions; and the proposed model for CNCs incorporated with AA particles during EISA.

As proposed above, CNC suspensions were mixed with AAs at different proportions. The four AAs have distinct molecular structures (Figure 1). Glycine simply has a side chain of hydrogen atoms, which shows limited hydrophilicity, whereas serine has a hydroxymethyl group as side chain, with a higher hydrophilicity as compared to glycine [26]. On the other hand, phenylalanine and histidine, respectively, show hydrophobic side chains of aromatic ring and imidazole group. The hypothesized model for AA–CNCs interactions implies that the different side chains of AAs may localize on both hydrophilic and hydrophobic surfaces of cellulose crystals. Thus, the diverse structures of composed AAs may contribute to distinctions for the subsequent self-assembly behavior, especially for the chiral nematic pitch or the polarized directions.

The suspension of composed CNCs–glycine (G5) was observed by TEM. With the highest selected proportion of AA, the nanoparticles maintained the needle-like shape, similar to the pure CNCs. Focusing on the surface of the CNCs, there appeared to be some globular particles that were nearly 5–10 nm in diameter. This could be the aggregate for free glycine molecules, which tend to be adsorbed on the CNCs during the evaporation of water in the suspensions.

### 3.2. Optical Properties of the Composite Films

**Figure 2:**
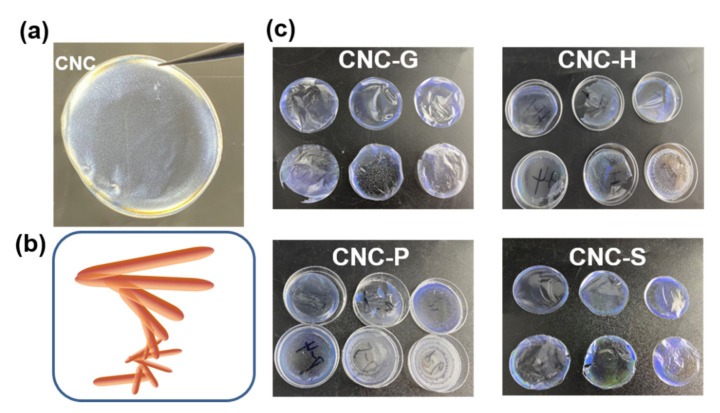
The image of (**a**) pure CNC film under POM, (**b**) model of chiral structure, and (**c**) photographs of CNC (top left) and corresponding composite films with different AA ratios.

Photographs of the composite films are presented in Figure 2. Under polarized light, pure CNCs showed an obviously golden color, especially on the edge of the film. The phenomenon is probably due to the “Coffee-Ring” effect of the negatively charged CNC nanoparticles [27]. This may also be caused by the disturbed drying kinetics transferred to the environment. Moving the films under visible light, the different iridescent colors were revealed. Pure CNC films were light blue, while the changing trends of the colors differed due to different samples. For the CNC-G and CNC-P groups, the blue color was less visible with the increase in AA ratios. However, for CNC-H and CNC-S, the colors tended to change to a longer wavelength region. In particular, the H5 sample presented a red color, and the S4 sample had a green edge. It is generally attributed to the successful intercalation of histidine and serine molecules, thus enlarging the pitch of the CNC layers. It can be hypothesized that the color changes rely greatly on the molecular structure of AAs. Glycine, which has the simplest side-chain structure, would have limited capability to vary the nanoparticles arrangements. Meanwhile, for phenylalanine, the aromatic rings may provide steric hindrance for intercalation into the layer gaps, hindering the formation of cholesteric structure [28].

**Figure 3:**
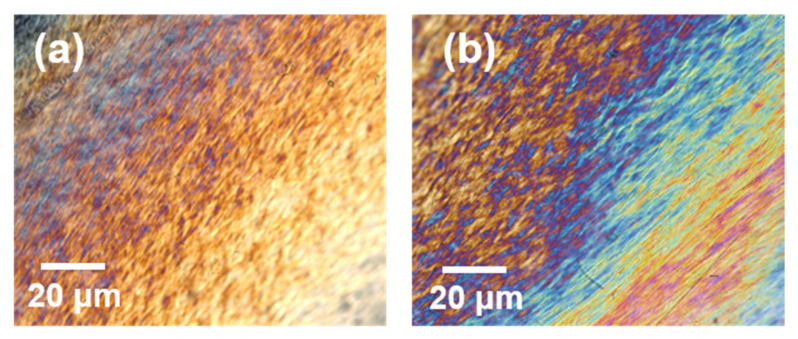
POM observations of the pure CNC film: (**a**) the center region and (**b**) the edge region.

The POM images provide deeper information for the understanding of the chiral nematic structures (Figure 3). The pure CNCs present perfect birefringence under the polarized light. The “fingerprint” textures are also observed, from which the pitch can be clearly determined. By comparing the center region and the edge region of the POM images, the “Coffee-Ring” effect is further proved. The changed gaps between the layers were the main reasons for the different colors under the visible light.

**Figure 4:**
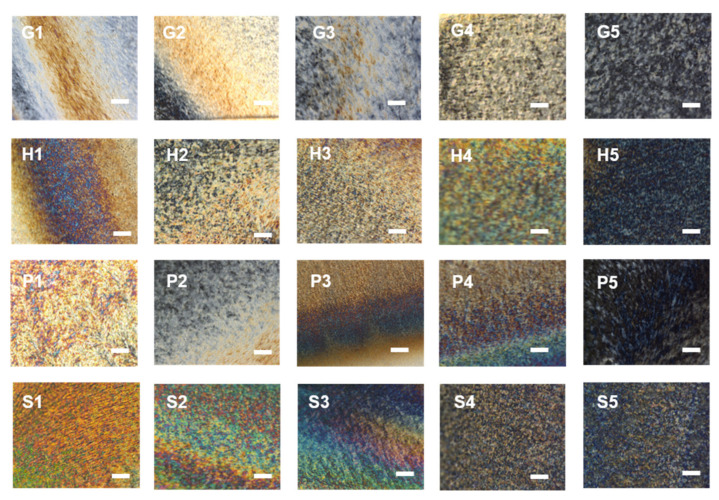
POM observations of the CNCs-based films incorporated with glycine, histidine, phenylalanine, and serine, with different AAs additions. Scale bar = 20 μm.

The POM images of all the composite films are shown in Figure 4. Polarized optical micrographs were all recorded at the center of the CNC films to avoid edge effects. Compared to pure CNC images, the fingerprints were disturbed after the combination of AAs. Glycine in particular had the least effect on the texture and birefringence of the films, with the obvious gaps indicating the pitch value. It is well-known that the pitch values are relevant to both the visible colors and POM images, which are easily affected by the addition of fillers [16]. For the other three groups of AA-added films, the fingerprint textures were affected. The changes of orientations for the domains are qualitatively indicative of less order, despite the presence of more colors for CNC-P and CNC-S samples. With the increasing amount of AAs added, the oriented textures were even less visible. The phenomenon is in keeping with previous reports on the CNCs orders with tuned tactoids annealing [29]. The darker POM images would be the result of the reduced concentrations in the dispersion mixtures, which are even below the critical CNC concentration (3 wt.%) for tactoid formation [30,31]. Furthermore, the birefringence observed by POM suggested that CNC-H and CNC-P with high AA concentrations (H5, P5) became more isotropic, though there remained some oriented domains together with the wider layer gap indicated from visible photographs. The hydrophobic side chains of histidine and phenylalanine may play critical roles during interactions with CNCs and the following evaporation.

**Figure 5:**
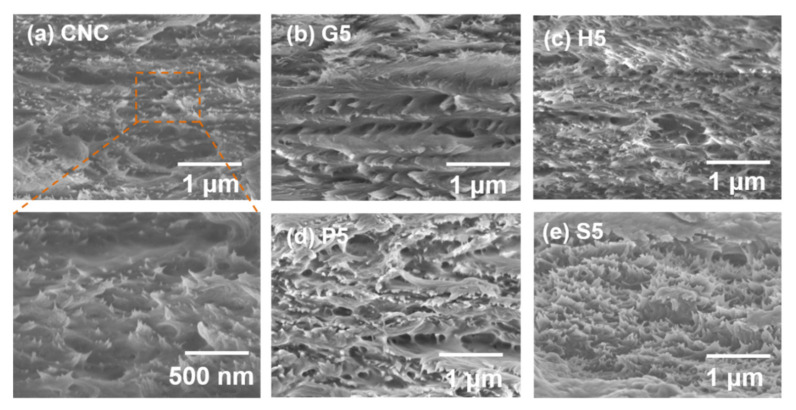
SEM images of the CNC-based films incorporated with the highest proportion of (**a**) CNC (with the enlarged image on bottom left), (**b**) glycine (G5), (**c**) histidine (H5), (**d**) phenylalanine (P5), and (**e**) serine (S5).

To investigate the morphology and the chiral nematic arrangements of the nanoparticles, the cross-sections of the composite films were observed in SEM images (Figure 5). Interestingly, pure CNC films present well-arranged layered structures, although the gaps were not as notable compared to the cross-sections of other composite films. Comparatively, G5 had an obvious cholesteric structure with a larger pitch than CNC film. The result of SEM observation agreed with the above distinctive images of POM characterization. The rougher surface was observed in three other types of composite films, though the gaps were not as large as for the G5 sample. The particles may be the remaining crystals of AAs, especially for hydrophobic histidine (H5) and phenylalanine (P5) [23]. The S5 sample exhibited a distinctive cholesteric structure with more needles heading in the perpendicular direction. Serine has been proved to be the easiest to intercalate into layered CNCs, which may reduce the electrostatic repulsion, allowing closer CNC packing [32,33,34]. Thus, the different mechanisms of CNC–AAs interactions contributed to diverse microstructural arrangements of the nanoparticles, which, in turn, provided the featured optical properties of the composite films.

**Figure 6:**
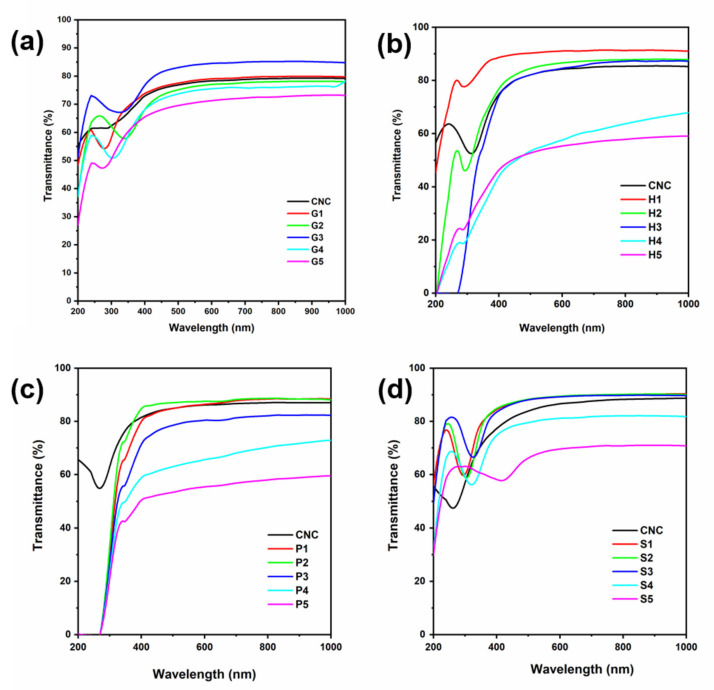
UV–Vis spectra of CNC film and composite films with addition of (**a**) glycine, (**b**) histidine, (**c**) phenylalanine, and (**d**) serine.

As we all know, the birefringence of the films as well as other optical properties can be qualitatively revealed by UV–Vis spectra (Figure 6). In each group, pure CNC films present similar spectra with the transmittance higher than 80%. There appeared a light absorbance in the region of 280 nm. The iridescence of blue color as observed under visible light was not revealed in the spectra, probably due to the determination mainly on the center of the CNC film rather than the edge region. The cooperation of AAs affected the arrangements of the cholesteric structure, which also provided various characterized peaks. For instance, there appeared a disturbance for the H3 sample in the region of approximately 600 nm, corresponding to the light green color in Figure 2. The disturbance was also observed for the P3 sample. Interestingly, the increase in histidine or phenylalanine had a limited effect on enlarging the pitch or altering the color reflection wavelength. It may be due to the decreased CNC concentration. Moreover, irregular aggregation and the reduced surface charge were both possible reasons contributing to the less ordered domains and blue-shift of the reflection peak [35,36]. Comparatively, the CNC-S group had more remarkable red-shift variations on the reflection wavelength (Figure 6d). This is in keeping with the above results on the well-dispersed CNC/serine mixture. It meanwhile favored the subsequent irregular cholesteric assembly. As a result, serine tended to orderly intercalate into the gaps, taking up the space and enlarging the pitch of the CNC-S films.

Although the CNC-H and CNC-P groups showed the critical requirements on concentration ratios for pitch tuning, it is interesting that the two groups of samples exhibit a good capability for UV-shielding. The phenomenon is typical for H3 and all the CNC-P samples. This is mainly because the imidazole group of histidine and the aromatic group of phenylalanine were more effective for UV adsorption [37,38]. CNC-P films had 100% blocking in the UVC region, while CNC-H preferred to absorb UVB and UVA light at a rate of more than 60%. However, the capability was achieved by the addition of more than 15% of histidine. This finding may provide a new perspective on the preparation of CNC-based films with multi-optical functions.

**Figure 7:**
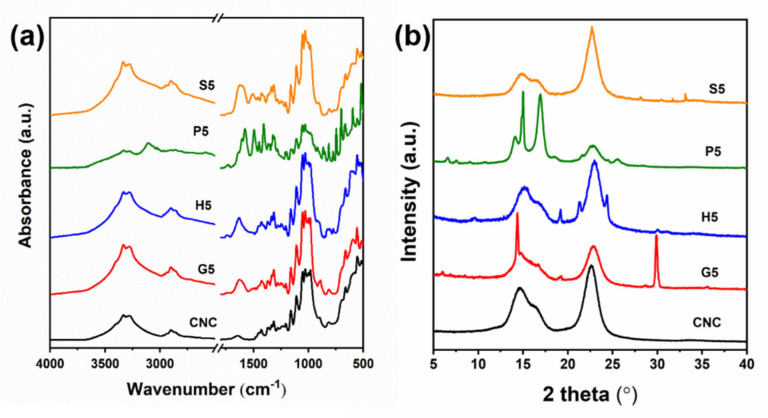
ATR-IR spectra (**a**) and XRD patterns (**b**) of CNC film and the composite films with the highest addition proportion of AAs.

A series of optical and microstructural observations have proved the distinctive applicable capabilities for the films. The inner chemical linkages and functional groups in the composite films were recorded by ATR-IR (Figure 7a). All the films present typical peaks of cellulose, such as the sharp peaks at regions 1030 cm^−1^ to 1060 cm^−1^ that correspond to the C–O stretching vibrations of alcohols in the composite materials. A similar peak appeared at 1096 cm^−1^ due to the CH2 vibration of cellulose Iβ [39]. The incorporation of AAs was proved by the wider bands at approximately 3300–3500 cm^−1^, which is ascribed to the stretching vibrations of –NH2 and –OH. More notably, the broader and higher peaks at the 1580–1720 cm^−1^ region indicated the considerable amount of amides introduced to the materials [32]. However, in this area, the S5 sample showed overlapped peaks compared to the other samples, probably due to the hydroxyl side chains interacting with amides by hydrogen bond linkages, whereas for P5, a sharp peak with shoulders appeared, mainly caused by the large amount of aromatic groups located at 1650 cm^−1^ [40]. The typical band for P5 was also observed at 3100 cm^−1^, corresponding to –CH stretching vibrations of substituted benzene. As a result, the band in the high wavelength region (3300–3500 cm^−1^) indicative of hydroxyl groups was decreased for the CNC-P sample.

In addition to the chemical variations caused by the interactions of CNCs and AAs, crystal structural changes of composites films were revealed by XRD (Figure 7b). Pure CNC film presented typical patterns of cellulose, with peaks at 14.8°, 16.5°, and 22.5°, respectively, assigned to (1–10), (110), and (200) lattice planes, with the high crystallinity of 74.8% [41]. It was noted that the two hydrophilic lattice planes of (1–10) and (110) exhibited uneven peak heights. This is mainly due to the preferred orientation of the cellulose crystals, especially for the cholesteric structure of the films induced by EISA [42]. At the region of approximately 2θ = 34.5°, due to the crystal length along the cellulose chains’ direction, the peaks were negligible for all the samples, which is uncommon for crystalline cellulose. It suggested that the recrystallization of EISA-induced films mainly took place in the direction perpendicular to the cellulose crystalline chains. Incorporation of the AAs introduced some new sharp crystalline peaks in the patterns. The phenomenon is particular for composite films combined with either glycine, histidine, or phenylalanine, respectively, which contained the characterized peaks for the corresponding AAs. However, for the S5 sample, the peaks for crystallized serine were not obvious, indicating the good interaction of serine with cellulose crystals. The intercalation of highly hydrophilic serine probably took place on the (1–10) and (110) planes of cellulose crystals, which can be strongly supported by the lowered peak height and the enlarged peak width compared to the patterns of pure CNC.

## 4. Conclusions

In the present work, chiral nematic CNC films individually composited with four types of AAs (glycine, histidine, phenylalanine, and serine) were easily prepared by evaporation-induced self-assembly. Iridescence and birefringence phenomena of the films were successfully observed. Chemical structural characterization proved the intercalation of the AAs into layers of cellulose crystals. The intercalations disturbed the morphologies on the cross-sections of the films, enlarging the pitch values and contributing to the trend of red-shift of the reflective wavelength. Among the AAs, serine presented the most compatible intercalation into cellulose crystals. Meanwhile, histidine and phenylalanine composites films showed high shielding capabilities of UV light in diverse wavelength regions. The proposed preparation for chiral nematic CNC films with tunable optical properties may provide new strategies for multi-functional sustainable materials.
